# The effect of a smartphone application on women’s performance and health beliefs about breast self-examination: a quasi-experimental study

**DOI:** 10.1186/s12911-021-01609-4

**Published:** 2021-08-24

**Authors:** Mitra Shakery, Manoosh Mehrabi, Zahra Khademian

**Affiliations:** 1 Department of Nursing, School of Nursing and Midwifery, Shiraz University of Medical Sciences, Shiraz, Iran; 2 Department of E-Learning in Medical Sciences, Virtual School, Shiraz University of Medical Sciences, Shiraz, Iran; 3 Community Based Psychiatric Care Research Center, Department of Nursing, School of Nursing and Midwifery, Shiraz University of Medical Sciences, Shiraz, Iran

**Keywords:** Breast neoplasms, Breast self-examination, Cell phone, Health behavior, Health belief model, Telemedicine

## Abstract

**Background:**

Breast Self-Examination (BSE) is a simple and inexpensive method for early diagnosis of breast cancer. This study aimed to determine the effect of a smartphone application on women’s performance and health beliefs regarding BSE.

**Methods:**

In this quasi-experimental study, 150 women referring to therapeutic clinics in Jahrom, Iran from December 2019 to May 2020 were randomly assigned to an intervention or a control group. The intervention group participants had access to a smartphone application including BSE reminder, training, alarm, and feedback to the therapist. The application also contained educational movies and self-assessment. The study data were collected using Champion’s Health Belief Model Scale and BSE information record form before and six months after the intervention. Then, the data were entered into the SPSS 21 software and were analyzed using descriptive statistics, paired t-test, independent t-test, Chi-square, ANCOVA, Mann–Whitney, and Wilcoxon tests.

**Results:**

After the intervention, the largest number of BSEs was four times among 60% of the participants in the intervention group and once among 24% of the participants in the control group during four months (p = 0.001). After the intervention, the mean differences of the scores of perceived susceptibility (1.03 ± 2.65 vs. 0.01 ± 0.42, p = 0.001), BSE barriers (2.80 ± 5.32 vs.  0.04 ± 1.43, p = 0.001), self-efficacy (10.75 ± 7.63 vs. − 2.75 ± 2.44, p = 0.001), and health motivation (2.77 ± 3.70 vs. − 0.29 ± 0.63, p = 0.001) were significantly higher in the intervention group compared to the control group. However, no significant difference was observed between the two groups with regard to perceived severity and BSE benefits after the intervention.

**Conclusions:**

Access to the smartphone application enhanced the participants’ performance and health beliefs regarding BSE in the areas of perceived susceptibility, self-efficacy, and health motivation. Therefore, we recommend using the same smartphone application to improve women’s performance and health beliefs regarding BSE.

**Supplementary Information:**

The online version contains supplementary material available at 10.1186/s12911-021-01609-4.

## Background

Breast cancer, as a non-communicable and chronic disease, is highly prevalent among females [1, 2]. With 2.3 million new cases and nearly 685 thousand new deaths, it was the most common cancer diagnosed and the fourth leading cause of cancer death in 2020 [2]. In Iran also, breast cancer accounted for almost 34% of cancers among females [3]. However, it has been reported that breast cancer occurred one decade earlier among Iranian women compared to those living in Western countries [4].

Since the occurrence of breast cancer cannot be completely prevented, the most appropriate method for coping with this disease and preventing its undesirable consequences is early diagnosis and timely treatment [5]. It is believed that early detection of breast cancer and subsequent appropriate treatment can increase patients' survival rate in the long term [6]. Evidence has indicated that participation in breast cancer screening can reduce the mortality associated with breast cancer by 40% [7].

Breast Self-Examination (BSE) is a simple and inexpensive method for early diagnosis of breast cancer, and is a realistic method for early discovery of the disease in some countries, particularly developing ones [8, 9]. BSE refers to examining the breasts after the menstrual cycle when the breast tissue is soft and, consequently, abnormal tissues can be distinguished from normal ones [10]. Different statistics on the frequency of mass identification using BSE have been reported, with 60% among Mexican women and 25% among American women [9, 11]. Despite the importance of BSE as a breast cancer screening strategy in developing countries, women's awareness and performance of this behavior were low in African countries, South Asia, and Iran. [12, 13].

Educational programs play a significant role in improvement of women knowledge, beliefs and practice in regard to breast cancer screening [14]. Trainings based on the scientific models and theories, according to their conceptual framework, provide a purposeful instrument for empowerment of the target population for promotion of individuals' control on their own health [14, 15]. The Health Belief Model (HBM) is one of the most popular models used for prevention of diseases and identification of the effective factors in screening behaviors [14, 16]. The model assumes that preventive behaviors are based on individuals' beliefs. It was designed to predict which groups of people use preventive measures and which do not. Therefore, it suggests interventions to reduce people's reluctance to health care. The likelihood of individuals' taking action for health increases when they realize that they are at risk of a health problem (perceived susceptibility), the problem is serious enough to need an action (perceived severity), and the expected benefits of the practice outweigh the anticipated costs (perceived benefits). Additionally, they do not find the pursuit of a new behavior costly, painful, and unpleasant (perceived barriers), there are accelerating forces that motivate them to take action (perceived motivation or cues to action), and they sense their ability to take the actions required (self–efficacy) [17–19]. The educational programs designed based on the HBM have shown a considerable impact on the promotion of breast cancer preventive behaviors among medical staff [20]. In another study, self-efficacy and perceived susceptibility were the major predictors of BSE performance among nursing students [21].

Electronic learning techniques such as teaching and learning using smartphones have been highly developed in recent years [22]. Previous studies suggested the use of smartphones in promoting health knowledge, behaviors, and outcomes [23, 24]. Using smartphone applications has been reported to have a positive effect on the performance of BSE among females [25]. Health education based on the novel educational techniques in the field of BSE can enrich the quality of education, increase women's awareness regarding BSE, and change their attitudes and scientific skills, so that they accept BSE as a health habit [26]. However, one of the factors preventing women from BSE has been found to be forgetting the appropriate time for this behavior [27]. Since people spend a lot of time using their smartphones and the issues learned through the applications including educational films remain well in the memory, the interventions that educate individuals and remind them about the appropriate time for BSE may exert positive impacts on their performances and beliefs. Nevertheless, scarce studies have been conducted on the effects of such interventions on women's performances and health beliefs. Therefore, the present study aimed to evaluate the effect of using a smartphone application on women's performance and health beliefs regarding BSE.

## Methods

### Design and participants

This quasi-experimental study with a controlled pre/posttest design was conducted on the women referred to Honari and Peymaniyeh clinics in Jahrom, Iran from December 2019 to May 2020. Based on a previous study [28] reporting the mean ± Standard Deviation (SD) of self-efficacy in the two groups as 3.4 ± 1.6 and 4.17 ± 1.07, considering α = 0.05 power of 90%, and effect size = 0.566 and using the G-power software, a 134-subject sample size was estimated for the research (n = 68 in each group). Considering a 10% probability of loss, the sample size was increased to 75 individuals in each group. The inclusion criteria of the study were being willing to take part in the research, signing the written informed consent form, not suffering from underlying breast disorders, having an Android smartphone, being able to use the smartphone for reading educational materials in applications, not having participated in BSE educational programs within the past year, and aging 18–60 years. The exclusion criteria of the study were not being able to continue cooperation in the research, suffering from severe physical and mental problems, taking part in educational programs related to BSE in the course of the study, and not completing the study instruments. Totally, 150 eligible women were selected by simple random sampling using the table of random numbers. Then, they were divided into an intervention (n = 75) and a control (n = 75) group via block randomization using the Random Allocation Software (block size of four). It should be noted that randomization was carried out by an expert who was unaware of the study groups. Then, the sample selection sequences were placed in numbered and sealed envelopes. After the beginning of the research, ten individuals in the intervention group were excluded due to unwillingness to participate in the study (n = 3), excessive stress while BSE (n = 2), and inability to install the application due to Android versions below 4.0 (n = 5). Thus, the number of participants in the intervention group decreased to 65 (Additional file 1).

### Development of smartphone application

At first, the educational materials related to BSE, prevention of cancer, and self-examination questions were extracted from reliable resources and were scientifically evaluated by two experts in community health nursing and a gynecologist. Then, the materials were revised and the educational movies were made in the form of animations. After that, the materials were prepared in the form of scenarios (adjusting guidelines and changing lessons to electronic formats) with the cooperation of electronic content production experts and images, animations, and audio and video clips were added. Afterwards, the materials were prepared in the form of installable applications on electronic devices using Android Studio, which is a programming environment for the Android platform. These applications were then evaluated by technicians and electronic content production experts. After all, the BSE application including two parts, i.e., “BSE” and “breast cancer educational materials” (with the sizes about 30–39 Megabytes for each part), was created. Both applications could be installed on smart electronic devices and could be used online. The enter key, about us, and help were there on the main page, and the users could move between the pages and use the contents by selecting them. It should be mentioned that installation of the application required connection to the internet.

In the next stage, the designed application was installed on the intervention group participants' smartphones and its usages were explained. The application was tested for a month, and the participants were asked to contact the researcher in case they had any problems or questions regarding working with the application.

### Intervention

The intervention group participants had access to the BSE smartphone application for six months, but the control group received no interventions and just referred to the clinics regularly. We had planned the intervention to last four months, however, due to the non-payment of the hotspot cost by the application development company, the problem in recording the results of the BSE performance occurred for the second two months. For this reason, we asked the participants to continue the study for another two months. Therefore, the intervention period lasted for 6 months.

The BSE application included an alarm system, a reminder (in form of a text message), a video clip training BSE accurately, four videos about breast cancer, and feedback to the therapist. The reminder prompted the participants to perform BSE based on their menstrual cycles. For the participants with regular menstrual cycles, BSE had to be performed on days 8–10 of the menstrual cycle considering the first day of the cycle. For those with irregular menstrual cycles as well as for menopausal women, BSE had to be performed on a fixed day every month. After installing the application, the participants were required to insert their demographic information as well as their menstruation date, and then, the exact date of BSE performance was computed by the application. Alarms were sent a day before the due date, at the due date, and a day after that. The reminder included an alarm ring, a pink sign on top of the smartphone, and a text message (Today, you should perform BSE). After BSE performance, no alarms were sent on other days. After confirmation of the reminder, the participants could watch an educational movie containing the step-by-step BSE technique in both standing and lying down positions according to their selected examination method. Then, they were required to answer questions regarding the existence of a palpable mass, abnormal discharges, nipple retraction, breast lumps, inflammation, color change, and existence of ulcers on the breast skin and to send the responses to the researcher via the feedback system. Each month, a video related to breast cancer educational materials (risk factors, breast pain and discharge, and prevention) was shown to the participants. This was possible after they confirmed watching the BSE educational movie and reporting the results. At the end of the second section, the participants were asked questions regarding breast cancer educational materials in order to improve their learning. It should be noted that the application's feedback system immediately transferred the participants' information to the researcher.

### Data collection

The study data were collected using a demographic information form, Champion's Health Belief Model Scale (1984), and self-examination record form. The demographic form included questions about age, education level, marital status, living place, and household's average monthly income. Furthermore, the form included questions regarding information about breast cancer, and source of the information, BSE performance, history of medical breast examination, or mammography, and family history of breast cancer.

Champion's Health Belief Model Scale was designed based on the constructs of the HBM and explored women's beliefs regarding breast cancer screening methods based on this model's constructs and variables [29]. The questionnaire items were divided into two sections, namely BSE and mammography. However, only the first part was used in the present investigation. The items could be responded via a five-option Likert scale ranging from one (completely disagree) to five (completely agree). This part aimed at assessment of the participants' health beliefs in six dimensions. Perceived susceptibility (individuals' perception of vulnerability to cancer) included three questions whose scores ranged from 3 to 15. Perceived severity (individuals' perception of the danger and seriousness of the disease complications) contained seven questions, with scores ranging from 7 to 35. The benefits of BSE (positive outcomes of disease prevention) were assessed by six questions and the scores could range from 6 to 30. Barriers of BSE (the factors preventing BSE) included nine questions whose scores could range from 9 to 45. Self-efficacy (individuals' confidence in their ability to successfully perform the behavior) was evaluated using 10 questions and the scores ranged from 10 to 50. Finally, health motivation (health-related beliefs) was explored via nine questions and the scores could range from 9 to 45. In the perceived barriers, higher scores represented negative attitudes. In other dimensions, however, higher scores indicated positive attitudes. Champion et al. designed this scale in 1984 and reviewed it in 1999. In that study, the construct validity of the scale was confirmed by confirmatory factor analysis and theoretical hypotheses testing. In addition, its reliability was approved by Cronbach's alpha = 0.65–0.90. Its test–retest reliability was also 0.40–0.68 [29]. In Iran, Teimouri et al. translated the scale using the standard multi-stage front-to-back technique. In that study, the validity and cultural appropriateness of the scale were verified by a group of gynecologists, health education specialists, physiologists, and nursing experts. The reliability of the scale was also assessed in a pilot study on 25 participants, revealing Cronbach's alpha > 0.7 [19]. In addition, in the current study, the reliability of the questionnaire was confirmed. Cronbach's alpha was calculated for the instrument perceived susceptibility (0.841), perceived severity (0.796), BSE benefits (0.776), BSE barriers (0.787), health motivation (0.735), and self-efficacy (0.758).

The self-examination record form was included in the application. It contained questions about BSE performance and some self-examination questions about breast cancer educational materials. The responses to the questions were recorded in the feedback system and were immediately transferred to the researcher. The feedback was coded based on the participants' national identity number.

Prior to the intervention, the participants in both study groups were requested to complete the questionnaires in the conference halls of Honari and Peimaniyeh clinics. After six months, due to the spread of COVID-19, the researcher (first author) went door-to-door and asked the participants to complete the questionnaires.

### Data analysis

Data analysis was done using the SPSS 21 software. The data were described by descriptive statistics. Independent t-test or Mann–Whitney test was used to compare the two study groups with respect to the scores of health beliefs and their dimensions. In addition, paired t-test or Wilcoxon test was utilized for within-group comparisons before and after the intervention. Because the p-value for between group comparison of marital status was borderline (p = 0.055), ANCOVA was employed for comparison of the two groups concerning the study variables after elimination of the effect of marital status [30]. P < 0.05 was considered statistically significant.

## Results

The total mean age of the study participants was 36.9 ± 10.5. All participants were residents of the city. The two groups were similar regarding demographic variables, information about breast cancer, and source of this information (Table 1).

**Table 1:** Distribution of the demographic variables, basic health information, and BSE information in the intervention and control groups

Group variable	Control (n = 75)		Intervention (n = 65)		Total		P-value*
	Number	Percent	Number	Percent	Number	Percent	
Age (years)							
18–30	17	22.7	22	33.8	39	27.9	0.336
31–50	48	64	36	55.4	84	60	
51–60	10	13.3	7	10.8	17	12.1	
Education level							
Below diploma	17	22.7	8	12.3	25	17.9	
Diploma	16	21.3	15	23.1	31	22.1	
Associate degree	9	12	9	13.8	18	12.9	
Bachelor's and higher degrees	33	44	33	50.8	66	47.1	0.463
Marital status							
Married	65	86.7	48	73.8	113	80.7	0.055
Single	10	9.7	17	26.2	27	19.3	
Household's average monthly income							
> 20 million Iranian Rials	18	24	13	20	31	22.1	
							0.57
≤ 20 million Iranian Rials	57	76	52	80	109	77.9	
Information about breast cancer							
Very low	15	20	14	21.5	29	20.7	0.926
Low	23	30.7	22	33.8	45	32.1	
Moderate	32	42.7	26	40	58	41.4	
High	5	6.7	3	4.6	8	5.7	
Source of information about breast cancer							
Studying	16	21.3	18	27.7	34	24.3	
Mass media	34	45.3	25	38.5	59	42.1	
Health staff and scientific meetings	13	17.3	14	21.5	27	19.3	
Information exchange with acquaintances	12	16	8	10.3	20	14.3	0.655
BSE performance							
Regularly	1	1.3	0	0	1	0.7	
Sometimes	36	48	39	60	75	53.6	
Never	38	50.7	26	40	64	45.7	0.263
History of medical breast examination							
Yes	22	29.3	21	32.3	43	30.7	
No	53	70.7	44	67.7	97	69.3	
History of mammography							
Yes	13	17.3	7	10.8	20	14.3	0.704
No	62	82.7	58	89.2	120	85.7	
Family history of breast cancer							
Mother	0	0	2	3.1	2	1.4	
Sister	3	4	0	0	3	2.1	
Mother's mother	1	1.3	0	0	1	0.7	
Mother's sister	1	1.3	1	1.5	2	1.4	
Others	12	16	9	13.8	21	15	0.268
No family history	58	77.3	53	81.5	111	79.3	

*Chi-square test

Table shows that the two groups were homogeneous in terms of demographic variables, including age, education level, marital status, and income as well as information about breast cancer, source of the information, BSE performance, history of medical breast examination, or mammography, and family history of breast cancer

**Fig. 1:**
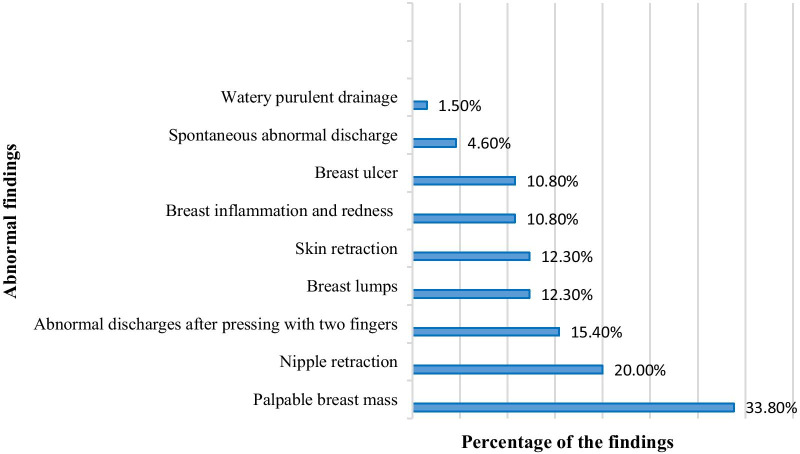
The frequency of abnormal findings in the intervention group, after intervention

The results showed no significant difference between the two groups concerning the performance of BSE before the intervention (p = 0.089). After the intervention, however, performance of BSE significantly increased in the intervention group compared to the control group (p = 0.001) (Table 2). According to the results, after the intervention the highest frequency of abnormal findings in the intervention group was related to the palpable breast mass (33.8%) and nipple retraction (20%) (Fig. 1).

**Table 2:** Comparison of the two groups regarding the frequency of BSE performance over a period of 4 months, before and after the intervention

Group variable	Control (n = 75)		Intervention (n = 65)		Total		P-value*
	Number	Percent	Number	Percent	Number	Percent	
Number of BSEs four months before the intervention							
0	46	61.3	32	49.2	78	55.7	0.089
1	17	22.7	23	35.4	40	28.6	
2	4	5.3	8	12.3	12	8.6	
3	6	8	2	3.1	8	5.7	
4	2	2.7	0	0	2	1.4	
Number of BSEs four months after the intervention							
0	57	76	0	0	57	40.7	0.001
1	18	24	0	0	18	12.9	
2	0	0	6	9.2	6	4.3	
3	0	0	20	30.8	20	14.3	
4	0	0	39	60	39	27.9	
P-value**	0.001		0.001	0.001			

*Chi-square test, ** Wilcoxon test

Table shows that before the intervention two groups were homogenous based on BSE performance over a period of 4 months. However, after the intervention, the largest number of BSEs was four times among 60% of the participants in the intervention group and once among 24% of the participants in the control group during four months

The results indicated no significant difference between the intervention and control groups with regard to health beliefs before the intervention. After the intervention, however, the mean differences of the scores of perceived susceptibility, self-efficacy, health motivation, and BSE barriers were significantly higher in the intervention group in comparison to the control group (p < 0.05). Nonetheless, no significant difference was observed between the two groups regarding the mean differences of the scores of perceived severity and BSE benefits (p > 0.05) (Table 3).

**Table 3:** Comparison of the mean scores of the dimensions of the health beliefs in the two groups before and after the intervention

Variable	Time	Control (n = 75)	Intervention (n = 65)	P-value
		Mean ± SD	Mean ± SD	
Perceived susceptibility	Before the intervention	12.08 ± 0.72	11.46 ± 2.58	0.129*
	After the intervention	12.09 ± 2.68	12.49 ± 2.19	0.445*
	Mean difference ***	0.01 ± 0.42	1.03 ± 2.65	0.001*
Perceived severity	Before the intervention	19.93 ± 5.68	19.71 ± 5.19	0.808**
	After the intervention	19.81 ± 5.75	21.35 ± 5.35	0.105**
	Mean difference***	− 0.12 ± 1.39	1.64 ± 5.24	0.078*
BSE benefits	Before the intervention	23.64 ± 3.36	23.43 ± 3.20	0.708**
	After the intervention	23.77 ± 3.37	24.06 ± 3.90	0.640**
	Mean difference***	0.13 ± 0.64	0.63 ± 4.61	0.076*
BSE barriers	Before the intervention	33.98 ± 4.90	34.53 ± 5.17	0.519**
	After the intervention	33.94 ± 4.84	37.33 ± 4.76	0.001**
	Mean difference***	− 0.04 ± 1.43	2.80 ± 5.32	0.001*
Self-efficacy	Before the intervention	26.27 ± 5.71	25.55 ± 5.88	0.469**
	After the intervention	23.52 ± 6.46	36.31 ± 7.62	0.001**
	Mean difference***	− 2.75 ± 2.44	10.75 ± 7.63	0.001*
Health motivation	Before the intervention	25.61 ± 4.55	25.74 ± 3.80	0.861**
	After the intervention	25.32 ± 4.49	28.51 ± 3.58	0.001**
	Mean difference***	− 0.29 ± 0.63	2.77 ± 3.70	0.001*

*Mann–Whitney test, ** Independent t-test, *** Post-test-pretest mean difference

Table shows that after the intervention, the mean differences of the perceived susceptibility, BSE barriers, self-efficacy, and health motivation were significantly higher in the intervention group compared to the control group. However, no significant difference was observed between the two groups with regard to perceived severity and BSE benefits after the intervention

## Discussion

The study results demonstrated that having access to the smartphone application enhanced the monthly performance of BSE among the intervention group participants. It also improved the intervention group participants' health beliefs in the areas of perceived susceptibility, self-efficacy, and health motivation. However, although these participants detected numerous abnormal findings following BSE, no significant improvement was observed in their health beliefs in the areas of perceived severity and BSE benefits compared to the control group. On the other hand, they reported higher perceived barriers.

The study findings revealed an increase in the performance of BSE after the intervention. This implied that reminding the participants about the time of performing BSE together with training BSE performance and other issues as well as reporting the results to the researcher encouraged the participants to perform this behavior. Some other interventions have also been found to be effective in increasing the performance of BSE among females. For instance, educational intervention based on the HBM constructs exerted positive effect on Iranian women [31]. Moreover, Heo et al. indicated that Android smartphone application including educational movies and reminder, enhanced the performance of BSE among the women aged below 30 years. In contrast to the present study, the application used in that study could not transfer feedback to the therapist. Besides, that study was conducted on a small number of participants in a single group [32].

In the current study, the designed application improved some health beliefs among the participants. For instance, it caused the women to perceive higher susceptibility towards BSE. In the studies carried out based on the HBM, the women who believed that they were prone to breast cancer and felt that they were at risk of this disease were more likely to perform screening behaviors [33, 34]. Furthermore, the present study findings revealed an improvement in the intervention group participants' health motivation compared to the control group. Similarly, another research showed that educational interventions increased health motivation in the area of breast cancer prevention behaviors [35].

In the present study, the educational intervention promoted the participants' self-efficacy in conducting prevention behaviors. Improvement of the participants' self-efficacy indicates that the smartphone application had assured the participants that they had the ability to perform BSE [17–19]. A Turkish study also confirmed that the women with higher self-efficacy were more likely to perform BSE compared to those with lower self-efficacy [36]. Another study has also demonstrated the effect of an educational intervention based on HBM on individuals' self-efficacy in BSE performance [35]. On the contrary, another study conducted in Iran showed that the educational intervention could not improve self-efficacy about breast cancer preventive behavior among female medical staff [37].

In the current research, the smartphone application was not effective in perceived severity. In contrast, some studies have reported that educational interventions were effective in improvement of the participants' belief in the severity of the consequences of breast cancer or their perceived severity. For instance, some studies carried out in Iran disclosed that the education based on HBM were effective in perceived severity [31, 38]. Perceived severity can act as a double-edged sword; in case of excessive perceived severity, ignorance or non-performance of preventive behaviors may occur [38]. This might be the reason for the increase in the performance of self-examination in the present study in spite of the fact that the intervention had no considerable effects on the perceived severity. Furthermore, some study participants detected abnormal findings after self-examination and reported them to the researcher, which could have played a role in following up the findings and reducing the participants' worries about the severity of the problem.

The current findings revealed that the smartphone application failed to improve individuals' perceived benefits of BSE. Contrary to this finding, some educational interventions in previous studies had improved the perceived benefits of women [31, 35]. This finding is surprising in the present study because women perceived the benefits of self-examination in a tangible way, as some abnormalities were reported in the intervention group, but these individuals still did not report a better perceived benefit of the BSE.

In the present study, the intervention increased the perceived barriers of BSE performance. Consistently, other studies indicated that educational interventions enhanced women's perception of BSE barriers [31, 35]. It is believed that in case women knew the screening behaviors, they would reduce the perceived barriers of these behaviors and do BSE [18, 38]. In the current research, involvement of the intervention group participants in BSE behaviors may have led them to perceive more barriers, some of the most important of which were shame, anxiety, and forgetting the appropriate time for performing the behavior [27, 39].

Overall, the educational intervention in the present study improved health beliefs in the areas of perceived susceptibility, health motivation, and self-efficacy, but not in perceived severity and perceived benefits. Lack of improvement in some beliefs might be attributed to the fact that the intervention was not designed based on HBM. However, it could affect some beliefs indirectly. Furthermore, although only three dimensions of health beliefs were significantly improved after the intervention, a significant improvement was observed in the individuals' performance of BSE. In other words, the participants were able to detect abnormal findings and report them to the researcher through the application. This could help healthcare providers follow up the identified problems and accelerate seeking for treatment.

It should be noted that although more than 40% of the participants had Bachelor's and higher degrees and more than 20% of them reported the history of breast cancer in their family members, almost half of them had never performed BSE prior to the intervention and most of them had moderate knowledge about breast cancer. This revealed the necessity to use universal interventions for increasing women's knowledge and performance in this regard.

The difference between the present study and those conducted in the past was the design of a Persian application based on the Iranian culture. Other specific aspects of the designed application included training BSE, reminding the appropriate time for performance of BSE, providing feedback to the researcher and presenting materials regarding the breast cancer prevention. The major strong points of the study were utilization of an application with unique features, consideration of a control group, and random allocation of the participants. Furthermore, due to the emergence of the COVID-19 pandemic and the limitations of holding face-to-face trainings or performing clinical breast examinations, using this smartphone application could be useful in educating and improving women's health beliefs and performance regarding BSE. Therefore, the use of such applications is recommended in the events such as the COVID-19 pandemic. However, the coincidence of the posttest with the COVID-19 pandemic imposed a limitation on the study because it could change the women's beliefs and health behaviors as well as their priorities in the field of healthcare. The other limitation of the study was that we did not evaluate the accuracy and quality of individuals' BSE performance. Therefore, we recommend that the accuracy of the BSE be examined in the future studies. Furthermore, extending the intervention phase for six months due to the application development company's failure to pay for the hotspot was another limitation of the current study.

## Conclusions

The study findings indicated that the educational intervention based on the smartphone application promoted the participants' BSE performance, perceived susceptibility, self-efficacy, and health motivation. However, it had no significant impacts on the perceived severity and benefits of BSE. On the other hand, it increased the perception of barriers. Hence, similar interventions are recommended to be used to improve women's performance and health beliefs in the field of BSE. Moreover, the constructs of the HBM are suggested to be used in designing applications in future investigations so as to enhance their effectiveness in health beliefs. Furthermore, it is necessary to train women to identify the real barriers of BSE performance and to find strategies to overcome them. Policymakers and healthcare providers also have to help promote women's health beliefs and screening behaviors by minimizing the barriers. Since the application used in the present study provided the users with the opportunity to immediately report their abnormal findings to the healthcare providers, similar applications are recommended to be used for gaining information about the breast health status of the women, particularly those living in remote regions.

## Supplementary Information

**Additional file 1:** Consort diagram.

## Abbreviations

BSE: Breast self-examination
COVID-19: Coronavirus disease of 2019
HBM: Health belief model
SPSS: Statistical package for the social sciences

## Declarations

### Availability of data and materials

The dataset of the present study is available upon request. Applicants can send their request for study data to Ms. Mitra Shakeri via email: mitrashakery61@gmail.com.

## References

[1] Bray F, Ferlay J, Soerjomataram I, Siegel RL, Torre LA, Jemal A. Global cancer statistics 2018: GLOBOCAN estimates of incidence and mortality worldwide for 36 cancers in 185 countries. *CA Cancer J Clin *2018, 68(6):394–424. 10.3322/caac.21492 30207593

[2] Sung H, Ferlay J, Siegel RL: Global Cancer Statistics 2020: GLOBOCAN Estimates of Incidence and Mortality Worldwide for 36 Cancers in 185 Countries. *CA Cancer J Clin* 2021, 71(3):209–249. 10.3322/caac.21660 33538338

[3] Roshandel G, Ghanbari-Motlagh A, Partovipour E, Salavati F, Hasanpour-Heidari S, Mohammadi G, Khoshaabi M, Sadjadi A, Davanlou M, Tavangar SM (2019). Cancer incidence in Iran in 2014: results of the Iranian National Population-based Cancer Registry. Cancer Epidemiol.

[4] Harirchi I, Kolahdoozan S, Karbakhsh M, Chegini N, Mohseni S, Montazeri A, Momtahen A, Kashefi A, Ebrahimi M (2011). Twenty years of breast cancer in Iran: downstaging without a formal screening program. Ann Oncol.

[5] Ginsburg O, Yip C-H, Brooks A, Cabanes A, Caleffi M, Dunstan Yataco JA, Gyawali B, McCormack V, McLaughlin de Anderson M, Mehrotra R *et al*: Breast cancer early detection: A phased approach to implementation. *Cancer* 2020, 126(S10):2379–2393. 10.1002/cncr.32887 PMC7237065 32348566

[6] Puliti D, Zappa M (2012). Breast cancer screening: are we seeing the benefit?. BMC Med.

[7] Seely JM, Alhassan T (2018). Screening for breast cancer in 2018-what should we be doing today?. Curr Oncol (Toronto, Ont).

[8] Miri M, Moodi M, Miri MR, Sharifzadeh G, Norozi E: Factors Affecting Breast Self-examination Behavior in Housewives in Birjand Based on the Transtheoretical Model. *IJBD* 2020, 13(2):28–36.

[9] Leon-Rodriguez E, Molina-Calzada C, Rivera-Franco MM, Campos-Castro A (2017). Breast self-exam and patient interval associate with advanced breast cancer and treatment delay in Mexican women. Clin Transl Oncol.

[10] Didarloo A, Pourali R, Gharaaghaji R, Rahimi B (2014). Comparing the effect of three health education methods on the knowledge of health volunteers regarding breast self-examination. J Nurs Midwifery Urmia Univ Med Sci.

[11] Roth MY, Elmore JG, Yi-Frazier JP, Reisch LM, Oster NV, Miglioretti DL: Self-detection remains a key method of breast cancer detection for U.S. women. *J Women's Health (2002)* 2011, 20(8):1135–1139. 10.1089/jwh.2010.2493 PMC3153870 21675875

[12] Pengpid S, Peltzer K (2014). Knowledge, attitude and practice of breast self-examination among female university students from 24 low, middle income and emerging economy countries. APJCP.

[13] Mahmoudabadi M, Saeidifar A, Safizadeh H (2018). Breast cancer screening behavior among nurses in kerman teaching hospitals and its relationship with the health beliefs model scales. IJBD.

[14] Noman S, Shahar HK, Abdul Rahman H, Ismail S, Abdulwahid Al-Jaberi M, Azzani M (2021). The effectiveness of educational interventions on breast cancer screening uptake, knowledge, and beliefs among women: a systematic review. Int J Environ Res Public Health.

[15] Khademian Z, Kazemi Ara F, Gholamzadeh S (2020). The effect of self care education based on Orem’s nursing theory on quality of life and self-efficacy in patients with hypertension: a quasi-experimental study. Int J Commun Based Nurs Midwif.

[16] Saei Ghare Naz M, Simbar M, Rashidi Fakari F, Ghasemi V: Effects of Model-Based Interventions on Breast Cancer Screening Behavior of Women: a Systematic Review. *APJCP* 2018, 19(8):2031–2041. 10.22034/APJCP.2018.19.8.2031 PMC6171373 30139040

[17] Champion VL, Skinner CS (2008). The health belief model. Health Behav Health Educ Theory Res Pract.

[18] Kirag N, Kizilkaya M (2019). Application of the Champion Health Belief Model to determine beliefs and behaviors of Turkish women academicians regarding breast cancer screening: a cross sectional descriptive study. BMC Womens Health.

[19] Taymoori P, Berry T (2009). The validity and reliability of Champion's health belief model scale for breast cancer screening behaviors among Iranian women. Cancer Nurs.

[20] Kalan FK, Jalili Z, Zareban I, Shahraki PM, Bahrami M: Predictors of preventive behavior breast cancer based on health belief model in teachers of secondary schools of Zahedan city. *J Zabol Univ Med Sci Health Serv (J Rostamineh)* 2013, 5(3):47–56.

[21] Momenyan S, Rangraz Jedi M, Sanei Irani F, Adibi Garakhani Z, Sarvi F (2014). Prediction of breast self-examination in a sample of nursing and midwifery students Qom City using health belief model, Iran. Qom Univ Med Sci J.

[22] Hlodan O (2010). Mobile learning anytime, anywhere. Bioscience.

[23] Khademian F, Rezaee R, Pournik O (2020). Randomized controlled trial: the effects of short message service on mothers' oral health knowledge and practice. Commun Dent Health.

[24] Khademian F, Aslani A, Ravangard R, Bastani P, Nami M, Jafari P (2020). Efficacy of a web application for stress management among Iranian college students during COVID-19 outbreak: a study protocol for randomized controlled trials. Trials.

[25] Naserian N, Ansari S, Abedi P (2018). Comparison of training via short messages and group training on level of knowledge and practice of middle-aged women about breast cancer screening tests. J Cancer Educ.

[26] Absavaran M, Niknami S, Zareban I (2015). Effect of training through lecture and mobile phone on breast self-examination among nurses of Zabol Hospitals. Payesh.

[27] Torkizade S, Soltanian Z, Davaridolatabadi N. Awareness and practice of female nursing, midwifery and paramedicine students at hormozgan university of medical sciences in relation to the risk factors, prognosis and prevention of breast cancer in the second semester of 2015–16. J Res Med Dental Sci. 2017;

[28] Pourhaji F, Vahedian Shahroodi M, Esmaily H, Pourhaji F, Harooni J (2013). Effects of training program-based on Stage of change Model to promote Breast self-examination behavior. Avicenna J Nurs Midwifery Care.

[29] Champion VL, Scott CR (1997). Reliability and validity of breast cancer screening belief scales in African American women. Nurs Res.

[30] Verma J: Data analysis in management with SPSS software: Springer, Berlin; 2012.

[31] Farma KK, Jalili Z, Zareban I, Pour MS (2014). Effect of education on preventive behaviors of breast cancer in female teachers of guidance schools of Zahedan city based on health belief model. J Educ Health Promot.

[32] Heo J, Chun M, Lee KY, Oh Y-T, Noh OK, Park RW (2013). Effects of a smartphone application on breast self-examination: a feasibility study. Healthc Inform Res.

[33] Akhtari-Zavare M, Juni MH, Said SM, Ismail IZ, Latiff LA, Ataollahi Eshkoor S (2016). Result of randomized control trial to increase breast health awareness among young females in Malaysia. BMC Public Health.

[34] Pagkatipunan PMN (2018). Peer leaders and phone prompts: implications in the practice of breast care among college students. APJCP.

[35] Masoudiyekta L, Rezaei-Bayatiyani H, Dashtbozorgi B, Gheibizadeh M, Malehi AS, Moradi M (2018). Effect of education based on health belief model on the behavior of breast cancer screening in women. Asia Pac J Oncol Nurs.

[36] Yilmaz M, Guler G, Bekar M, Guler N (2011). Risk of breast cancer, health beliefs and screening behaviour among Turkish academic women and housewives. APJCP.

[37] Eskandari-Torbaghan A, Kalan-Farmanfarma K, Ansari-Moghaddam A, Zarei Z (2014). Improving breast cancer preventive behavior among female medical staff: the use of educational intervention based on health belief model. MJMS.

[38] Ghaffari M, Esfahani SN, Rakhshanderou S, Koukamari PH (2019). Evaluation of health belief model-based intervention on breast cancer screening behaviors among health volunteers. J Cancer Educ.

[39] Tabari F, Abbaszadeh R, Torabi S, Amini F (2017). Barriers of breast self-examination: a review study from Iranian researchers. Bali Med J.

