# Supplementary material for: The effect of a smartphone application on women’s performance and health beliefs about breast self-examination: a quasi-experimental study
Source: BMC Med Inform Decis Mak. 2021 Aug 24;21:248. doi: 10.1186/s12911-021-01609-4 (PMC8383252; doi:10.1186/s12911-021-01609-4)
Supplement: Supplementary file 1 — Additional file 1: Consort diagram. [file 12911_2021_1609_MOESM1_ESM.doc]

**
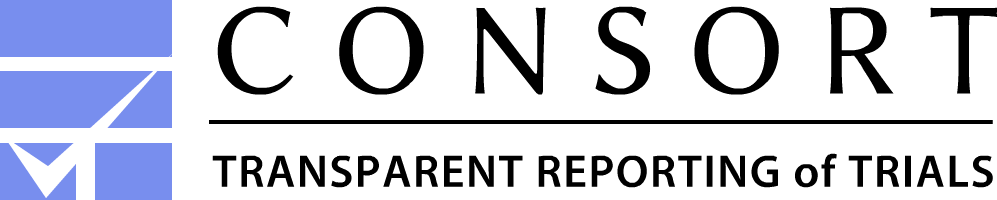
**

**CONSORT 2010 Flow Diagram**

**Allocation**

**Analysis**

**Follow-Up**

**Enrollment**

Assessed for eligibility (n= 150)

Excluded (n= 0)

♦  Not meeting inclusion criteria (n=0 )

♦  Declined to participate (n= 0 )

♦  Other reasons (n=0 )

Analysed (n=65)
♦ Excluded from analysis (give reasons) (n=0)

Lost to follow-up (give reasons) (n= 3)

Discontinued intervention (give reasons) (n= 7)

Allocated to intervention (n=75)

♦ Received allocated intervention (n=75)

♦ Did not receive allocated intervention (give reasons) (n= 0 )

Lost to follow-up (give reasons) (n= 0)

Allocated to control (n=75)

♦ Received allocated intervention (n=75)

♦ Did not receive allocated intervention (give reasons) (n=0 )

Analysed (n=75)
♦ Excluded from analysis (give reasons) (n= 0)

Randomized (n=150)

**Manuscript title:** The effect of a smartphone application on women’s performance and health beliefs about breast self-examination: A quasi-experimental study

**Authors:** Mitra shakery, Manoosh Mehrabi, Zahra Khademian
